# The 350th anniversary of scientific publishing: van Leeuwenhoek, the most prolific author of the *Philosophical Transactions of the Royal Society*

**DOI:** 10.1098/rsob.150044

**Published:** 2015-04-29

**Authors:** David M. Glover

**Affiliations:** FRS, Editor-in-Chief

This year marks the 350th anniversary of the launch of *Philosophical Transactions of the Royal Society*—the world's first scientific journal. This innovative way of recording the Royal Society's business proved to be a model for scientific publishing as we now know it. It provided a means of reviewing scientific discovery, recording it, and most importantly provided a means for disseminating these findings. The history of the Society's publishing enterprises is truly fascinating and can be accessed at https://royalsociety.org/publishing350/.

The celebration of this anniversary falls at a time when scientific publishing is in the midst of the revolution of electronic publishing. All of the Society's journals are now available online and some, such as *Open Biology*, are only available in this format. The Society's objectives are, even more than ever before, to make scientific findings freely available to the widest possible audience.

During the course of this year, we will commemorate some of the publications from the archives of *Philosophical Transactions* in the fields represented in *Open Biology*. We begin by celebrating the work of Antony van Leeuwenhoek, the most prolific contributor to *Philosophical Transactions*, which published over 100 of his letters to the Society. These concerned observations made through his microscopes of the most diverse biological subjects imaginable.

With van Leeuwenhoek's prolific observations, we witness perhaps the first example of how progress in biology has gone hand in hand with the development of technology; something very evident in the science of today. Here we wish to celebrate this duality and there can be no better way than by referring to one of van Leeuwenhoek's early pieces of correspondence. Out of his many letters, I have selected one that established him as the founder of microbiology [1]. I strongly recommend browsing this short article reproduced at http://rstl.royalsocietypublishing.org/content/12/133-142/821.full.pdf+html. You will see that the then editor of Philosophical Transactions and Secretary of the Royal Society, Henry Oldenburg, exercised much greater intervention than myself not only in translating van Leeuwenhoek's letters from the Dutch but also in making considerable editorial cuts.

In this issue of *Open Biology*, we would like to place seventeenth century microscopy into the context of technical developments that continue to drive our understanding of biology in the twenty-first century. We are very grateful to Mark Leake and his colleagues who have kindly reviewed the development of the light microscope over the intervening 300 or so years [2]. In a delightful parallel article in *Philosophical Transactions*, Nick Lane has taken us back to the long and prolific life of van Leeuwenhoek to recreate the scientific climate of the time and to place van Leeuwenhoek's pioneering works into their biological context [3].

We hope you enjoy reading both commentaries. You can access the complete archive of *Philosophical Transactions* at http://rstl.royalsocietypublishing.org/content/by/year.
